# Hybridization between alien species *Rumex obtusifolius* and closely related native vulnerable species *R. longifolius* in a mountain tourist destination

**DOI:** 10.1038/srep13898

**Published:** 2015-09-10

**Authors:** Koichi Takahashi, Masaaki Hanyu

**Affiliations:** 1 Department of Biology, Faculty of Science, Shinshu University, Matsumoto, Nagano 390-8621, Japan; 2 Institute of Mountain Science, Shinshu University, Asahi 3-1-1, Matsumoto, Nagano 390-8621, Japan; 3 Graduate School of Science and Technology, Shinshu University, Asahi 3-1-1, Matsumoto, Nagano 390-8621, Japan

## Abstract

Alien species expand their distribution by transportation network development. Hybridization between alien species *Rumex obtusifolius* and closely related native vulnerable species *R. longifolius* was examined in a mountain tourist destination in central Japan. The three taxa were morphologically identified in the field. Stem height and leaf area were greater in *R. longifolius* than *R. obtusifolius*; hybrids were intermediate between the two *Rumex* species. *R. longifolius* and the hybrids grew mainly in wet land and the river tributary; *R. obtusifolius* grew mainly at the roadside and in meadows. Hybrid germination rates of pollen and seeds were much lower than for the two *Rumex* species. Clustering analysis showed the three taxa each formed a cluster. Most hybrids were F1 generation; the possibility was low of introgression into the two *Rumex* species by backcross. This study clarified that (1) hybridization occurred between *R. obtusifolius* and *R. longifolius* because they occurred together in a small area, but grew in different water habitat conditions, and (2) hybridization was mostly F1 generation because hybrid pollen and seed fertility was low. However, we need caution about introgression into *R. longifoliu*s by *R. obtusifolius* in this area because of the slight possibility of F2 generation and backcrosses.

Alien plants invade many sites because of increasing human activities, such as development and tourism. They rapidly increased in Japan after the latter half of the nineteenth century, and today about one quarter of vascular plant species in Japan are alien and naturalized species^1^. Environmental changes by human disturbances induce invasion of alien plants^2^,^3^. For example, alien plants invade after a large-scale river development^4^ and often invade roadsides^5^,^6^. Invasion of alien plants possibly changes the ecosystem structure and function by occupying invasion sites and by suppressing native plants^7^,^8^. Sometimes alien plants reduce the growth environment of native plants, sometimes form interspecific hybrids between the alien plants and closely related native plants^9^,^10^, and not only form hybrids, but also cause introgression into native plants by backcross^11^,^12^. Interspecific hybrids may lead to extinction of native species^11^.

Alien plants spread to mountain areas because many people go there by the development of transportation networks^13^. Kamikochi in the Chubu-Sangaku National Park, central Japan, is a depositional plain formed by volcanic activity, and is an unusually wide plain at high altitude (about 1500 m above sea level, Figure S1). About 1.5 million people a year visit Kamikochi because it is a scenic area. Kamikochi is surrounded by many steep mountains, which induce many floods of rocks and mud. Therefore, erosion control work is considerable in Kamikochi. Currently, many alien plants are invading Kamikochi by tourists and vehicles^14^,^15^. In achieving a balance between maintaining the ecosystem and tourism resources, research on the invasion of alien plants and hybridization between alien and native plants is indispensable.

This study examined hybridization between alien species *Rumex obtusifolius* L. and the closely related native species *Rumex longifolius* DC. in Kamikochi. The two species are widely distributed in the northern Hemisphere^16^. *R. obtusifolius*, native to Europe, invaded the northern part of Japan at the end of the nineteenth century. Currently, the distribution of *R. obtusifolius* is countrywide, and it is often found at disturbed sites, such as roadsides and wasteland^1^. *R. obtusifolius* and *R. longifolius* mainly grow in dry and wet sites, respectively^1^,^17^. The number of *R. longifolius* is decreasing because of changes in habitat environments^18^, and is designated as a vulnerable species II^19^. The two *Rumex* species grow in Kamikochi, and the putative interspecific hybrids, based on the morphological traits, are found^14^.

Clarifying the ecological and morphological traits of parent species and their interspecific hybrids is important to understand the invasion of alien species and hybridization with native species^20^. As qualitative morphological traits of the two *Rumex* species, leaves and stems of *R. obtusifolius* are reddish and have hairs on the leaf abaxial side, unlike *R. longifolius*^17^,^21^,^22^. Hybrids between the two *Rumex* species are presumed to grow at locations adjacent to dry and wet sites. Whether F2 and later generations will remain in the future relates to the fertility of pollen and seeds of hybrids. If the germination rates of pollen and seeds of hybrids are high, hybridization progresses and genetic pollution spreads out. Therefore, investigating morphological traits, soil water conditions of habitats, and fertility of pollen and seeds of the two *Rumex* species and their hybrids is necessary to clarify the effects of alien species *R. obtusifolius* on native species *R. longifolius*.

If interspecific hybrid individuals between native and alien plant species have already backcrossed with the native species, introgression is presumed to occur, even for the putative native species individuals identified from the morphology^23^,^24^. Therefore, evaluating the spread of hybrids based on morphological traits only is difficult. The discrimination of hybrids is impossible unless we use molecular techniques. Especially, amplified fragment length polymorphism (AFLP) is often used for studies of hybridization^12^,^25^.

This study aimed to clarify if introgression into the native species *R. longifolius* by the alien species *R. obtusifolius* is occurring in Kamikochi, a mountain tourist destination, central Japan. For this purpose, we compared morphological and ecological traits among the two *Rumex* species and hybrids and identified the three taxa by using AFLP analysis.

## Results

We found total 193 reproductive individuals at the three plots (109 individuals of *R. obtusifolius*, 56 individuals of *R. longifolius* and 28 individuals of their hybrids, based on the morphological traits of fruits).

### Spatial distribution

**Figure 1:**
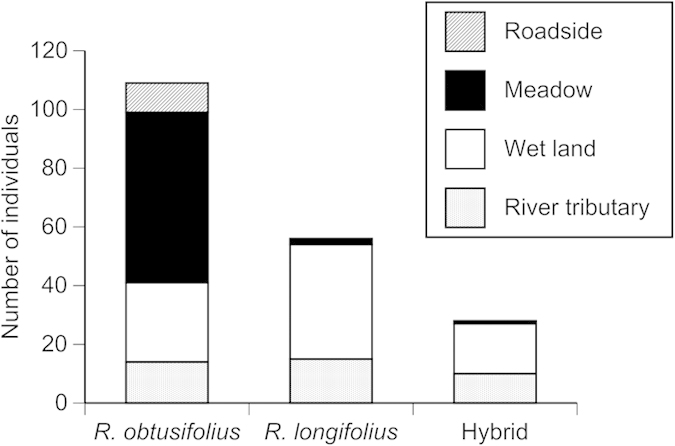
Numbers of individuals of *Rumex obtusifolius*, *R. longifolius* and the hybrid in habitat conditions of roadside, meadow, wet land and river tributary in one plot at Site 1 and two plots at Site 2 (total 0.12 ha) in Kamikochi, central Japan.

The three taxa grew in habitat conditions different from each other (χ^2^ = 71.7, d.f. = 6, *P* < 0.001, Fig. 1, S2). *R. obtusifolius* was distributed at various habitat conditions, such as roadside, meadow, wet land and river tributary. On the contrary, *R. longifolius* was distributed mainly in wet land and river tributary, and were hardly found in meadows and roadsides. The hybrids mainly grew in wet land and river tributary, like *R. longifolius*.

### Morphological traits

**Figure 2:**
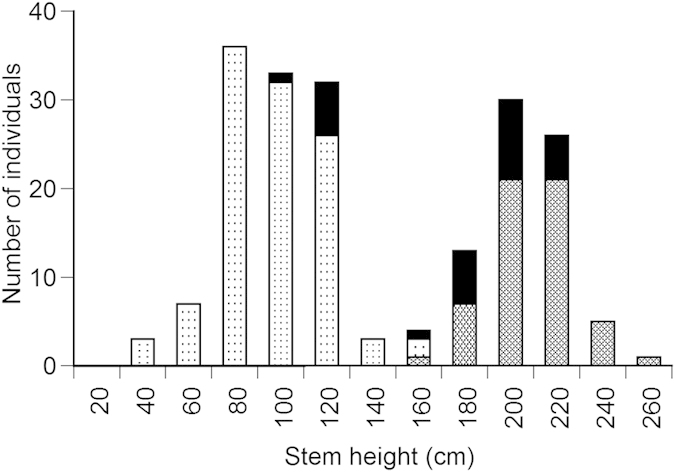
Frequency distributions of stem height for *Rumex obtusifolius*, *R. longifolius* and the hybrid in one plot at Site 1 and two plots at Site 2 (total 0.12 ha) in Kamikochi, central Japan. Stippling, cross-hatch shading and solid bars indicate *Rumex obtusifolius*, *R. longifolius* and the hybrid, respectively.

**Figure 3:**
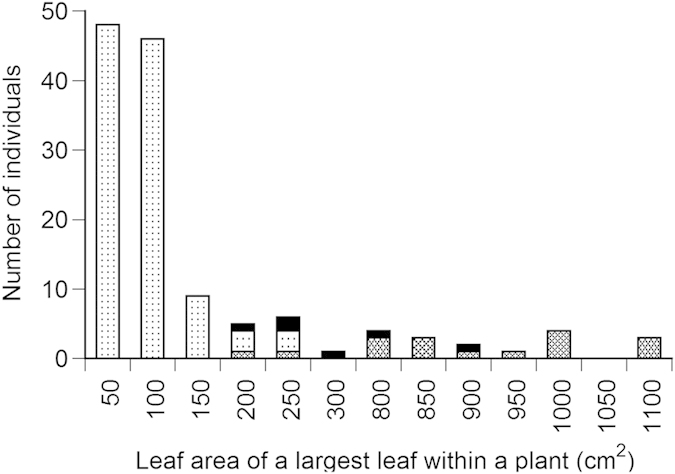
Frequency distributions of the leaf area of the largest leaf of a plant for *Rumex obtusifolius*, *R. longifolius* and the hybrid in one plot at Site 1 and two plots at Site 2 (total 0.12 ha) in Kamikochi, central Japan. Stippling, cross-hatch shading and solid bars indicate *Rumex obtusifolius*, *R. longifolius* and the hybrid, respectively.

The three taxa classified by the valves of fruits clearly differed in other qualitative and quantitative morphological traits. Many individuals of *R. obtusifolius* showed qualitative morphological traits of leaf hairs on the abaxial side, reddish stems and leaves, and no basal leaves (Table 1). By contrast, *R. longifolius* showed the opposite morphological patterns to *R. obtusifolius*, i.e., *R. longifolius* had basal leaves, but no leaf hairs and reddish color on stem and leaves. These qualitative morphological traits significantly differed among the three taxa (χ^2^-test, *P* < 0.001, Table 1), although these morphological traits were similar between *R. obtusifolius* and the hybrids. The three taxa also differed in stem height (Kruskal-Wallis test, *H* = 137.9, *P* < 0.001, Fig. 2), and leaf area of the largest leaf of a plant (Kruskal-Wallis test, *H* = 143.8, *P* < 0.001, Fig. 3). The stem height and leaf size of *R. longifolius* were greater than those of *R. obtusifolius*, and those of the hybrids were intermediate between *R. longifolius* and *R. obtusifolius*.

### Germination rates of pollen and seeds

The pollen germination rate of *R. obtusifolius* (47%) was significantly greater than that of *R. longifolius* (36%) because the 95% confidence intervals did not overlap between them (Figure S3). The hybrid pollen germination rate (3%) was considerably lower compared with *R. obtusifolius* and *R. longifolius*.

**Figure 4:**
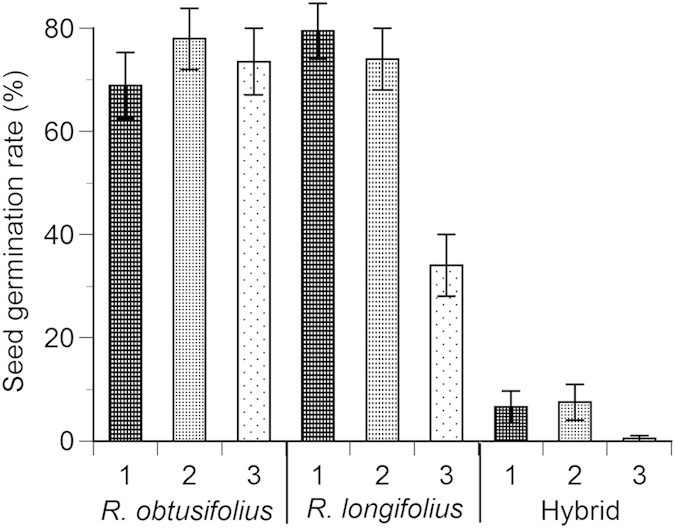
Seed germination rates (%) of *Rumex obtusifolius*, *R. longifolius* and the hybrid at 95% confidence intervals at water conditions of 1) complete flooding, 2) light flooding and 3) no flooding with wet filter papers.

The seed germination rate of *R. obtusifolius* was not significantly different among the three flood conditions (complete, light and no flooding), and ranged between 69% and 78% (Fig. 4). Although the seed germination rate of *R. longifolius* was 74% to 79% in complete and light flooding conditions, it decreased greatly to 34% in the no flooding condition. The seed germination rate of the hybrids was only 7% in the complete and light flooding conditions, and merely 0.5% in the no flooding condition.

### AFLP analysis

**Figure 5:**
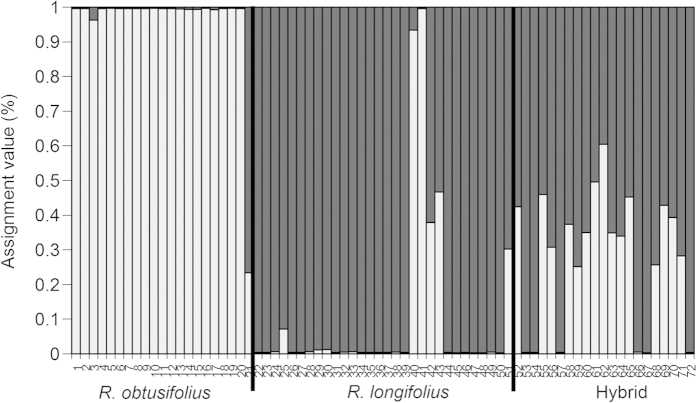
Bar plot of assignment proportions of individual plants (1–72) from the Structure analysis. Individual plants were morphologically identified as *Rumex obtusifoliu*s (1–21), *R. longifolius* (22–51) and the hybrid (52–72). The length of each bar reflects the Bayesian posterior probability that the 72 individuals belong to *R. obtusifoliu*s (light shaded part) and *R. longifolius* (dark shaded part).

We obtained 42 fragments by *Eco*RI-ACC/*Mse*I-CTA and 86 fragments by *Eco*RI-ACT/*Mse*I-CAT (total 128 fragments). The ∆*K* value was highest at *K* = 2, so that the *K* = 2 seemed to be plausible for clustering (Fig. 5, S4).

**Figure 6:**
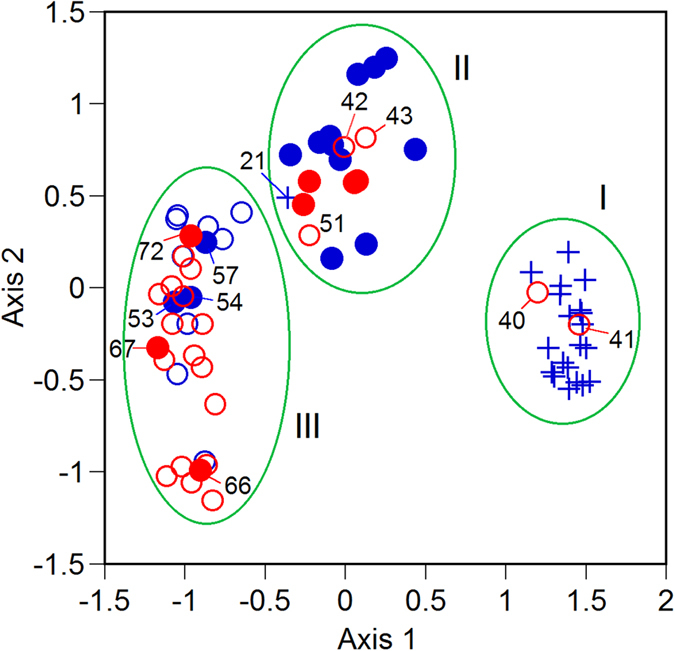
Principal coordinate analysis of morphologically identified plants as *Rumex obtusifoliu*s (cross), *R. longifolius* (open circle) and the hybrids (solid circle). Blue and red symbols indicate reproductive and non-reproductive individuals, respectively. Symbols with sample numbers are the individuals that are not included in the own cluster of the species. Three genetic clusters (circles marked as I, II and III) were supported by the cluster analysis with the complete linkage method.

Individuals were statistically classified into three clusters by principal coordinates analysis (PCoA) and clustering analysis from the AFLP genetic information (Fig. 6). The first and second axes explained 19.4% and 6.0% of the variations, respectively. Each morphologically identified taxon, including both reproductive and non-reproductive individuals, tended to be in the same cluster. Cluster II that included many hybrids was between cluster I of *R. obtusifolius* and cluster III of *R. longifolius*. However, one reproductive individual (No. 21) of *R. obtusifolius* was included in cluster II of the hybrids. Two non-reproductive individuals (Nos. 40, 41) and three non-reproductive individuals (Nos. 42, 43, 51) of *R. longifolius* were included in cluster I of *R. obtusifolius* and cluster II of hybrids, respectively. Three reproductive individuals (Nos. 53, 54, 57) and three non-reproductive individuals (Nos. 66, 67, 72) of the hybrids were included in cluster III of *R. longifolius*. Therefore, the three taxa, identified based on morphological traits, were not separated completely, although they formed their own clusters.

**Figure 7:**
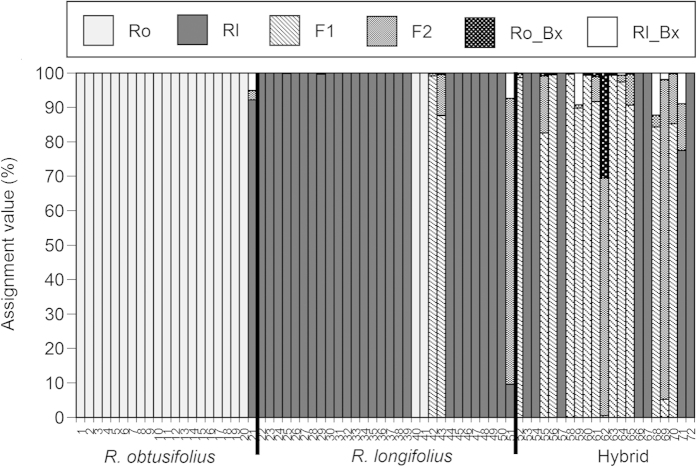
Bar plot of assignment proportions of individual plants (1–72) from the NewHybrids analysis. Individual plants were morphologically identified as *Rumex obtusifoliu*s (1–21), *R. longifolius* (22–51) and the hybrid (52–72). The length of each bar reflects the Bayesian posterior probability that the 72 individuals belong to *R. obtusifoliu*s (Ro), *R. longifolius* (Rl), F1, F2, backcross to *R. obtusifoliu*s (Ro_Bx) and backcross to *R. longifolius* (Rl_Bx).

Stochastic values assigned to each taxon were shown by NewHybrids analysis (Fig. 7). All *R. obtusifolius* individuals, except for one individual (No. 21), were assigned to *R. obtusifolius*. Individual No. 21 showed a possibility of backcrossing with *R. obtusifolius*. Some individuals of *R. longifolius* and the hybrids were assigned to a different taxon of the three taxa. Two individuals (Nos. 40 and 41) of *R. longifolius* were genetically *R. obtusifolius*, which agreed with the result of PCoA. Six hybrid individuals (Nos. 53, 54, 57, 66, 67 and 72), assigned to cluster III of *R. longifolius* by PCoA, were also assigned to *R. longifolius* by NewHybrids analysis. Similarly, two individuals (Nos. 42 and 43) of *R. longifolius* in cluster II of the hybrids were also assigned to the hybrids by NewHybrids analysis. Three individuals (Nos. 51, 62 and 69) of *R. longifolius* showed high assignment valves of the F2 generation; individual No. 62 among them showed 30.5% probability of backcrossing with *R. obtusifolius*.

## Discussion

*R. obtusifolius* tended to be discriminated from *R. longifolius* and the hybrids by the three clustering analyses (Structure, PCoA and NewHybrids), and the species identification was consistent between the morphological and genetic bases. By contrast, some non-reproductive individuals of *R. longifolius* differed from the result of the genetic analyses. Probably, the unclear discrimination keys, except for the valves and tubercles of mature fruits, caused this inconsistency. Although identification by mature fruits is clear, identifying *Rumex* species by the other morphological traits, such as by basal leaves, is difficult^26^. Therefore, species identification based on morphological traits other than mature fruits possibly causes misidentification of *Rumex* species. Especially for the hybrids, not only non-reproductive individuals but also reproductive individuals were included in the cluster of *R. longifolius*, i.e., these hybrids were genetically *R. longifolius*. This suggests that identification of the hybrids between the two *Rumex* species, based on morphological traits only, is difficult even for reproductive individuals.

Alien species *R. obtusifolius* grows in disturbed environments, such as roadsides, while native species *R. longifolius* grows mainly in wet lands and river tributaries. Many alien plant species adapt to dry and bright conditions^6^, and grow mainly in human-disturbed artificial sites^5^,^27^. Paved and unpaved roads are along both sides of Azusa River in Kamikochi for tourists and erosion control work. Therefore, disturbed sites appropriate for growth of *R. obtusifolius* are near river tributaries and wet lands in which *R. longifolius* grows. Seeds of alien plant species tend to be dispersed along roadsides^28^, and bright environments and removal of litter from the soil surface increase the distribution of alien plant species along roadsides^13^. Especially, seed germination of *R. obtusifolius* increases by bright conditions^29^. Therefore, *R. obtusifolius* tends to grow along roadsides, and a hybrid zone may be formed between the distribution sites of the two *Rumex* species if a road exists near wet lands and river tributaries in which *R. longifolius* grows.

The interspecific hybrids showed qualitative morphological traits similar to *R. obtusifolius* in terms of presence or absence of leaf hairs on the abaxial side, basal leaves and reddish color on leaves and stems. By contrast, the hybrids showed intermediate patterns between *R. obtusifolius* and *R. longifolius* in terms of the quantitative morphological traits stem height and leaf size. The leaf dimensions, a quantitative morphological trait, of hybrids between two *Salix* species are intermediate between the two *Salix* species, although the qualitative morphological traits of the hybrids are similar to those of one of the two *Salix* species^30^. Qualitative morphological traits are controlled by one or two genes whose expression appears to be largely dominant^31^, but quantitative morphological traits are considered to be controlled by multiple dominant genes^32^ and are affected by genetic variations due to backcross. Probably, the qualitative morphological traits of the interspecific hybrids in this study are also controlled by dominant genes of *R. obtusifolius*. Quantitative morphological traits of interspecific hybrids are intermediate between the parent species^33^. The number of chromosomes is 2*n* = 40 for *R. obtusifolius* and 2*n* = 80 for *R. longifolius*; the number of chromosomes of the interspecific hybrids is 2*n* = 60, intermediate between the parent species^22^, indicating that the quantitative morphological traits of the hybrids probably became intermediate.

The possibility of the existence of the F2 generation and backcrossed individuals is quite low because of the low germination rates of pollen and seed of the hybrids. This inference was supported by the genetic analyses, i.e., the hybrids were intermediate between the parent species by the PCoA and Structure analysis, indicating that hybrid individuals were almost F1 generation. The fertility of interspecific hybrids is quite low^34^. Therefore, the possibility of genetic pollution of the parent *Rumex* species is extremely low by interspecific hybridization.

At least 39 alien plant species, including *R. obtusifolius*, have been recognized in Kamikochi, a mountain tourist destination^15^. The invasion of alien species into Kamikochi by many tourists and vehicles can no longer be avoided because about 1.5 million people a year visit Kamikochi. Studies of the invasion and hybridization of alien plant species in the field is indispensable for compatibility between the maintenance of ecosystems and tourism. This study considered that the risk of hybridization between the two *Rumex* species for genetic pollution was not such a threat for the continuance of the genetic structure of each species. We presume that alien species *R. obtusifolius* will not exclude native species *R. longifolius* completely because the water habitat conditions required for its distribution differ between the two *Rumex* species. However, we recognized a low possibility of an F2 generation by NewHybrids analysis. Of individuals with the possibility of the F2 generation, one individual showed 30% of the possibility of a backcross. *R. obtusifolius* also crosses with other *Rumex* species, including *R. longifolius*. The F2 generation and backcrossed individuals have also been slightly recognized by other studies, although most hybrids were F1 generation^35^. Continuous development for tourism generates artificial environments, such as construction of buildings and roads that contribute to the spread of suitable environments for *R. obtusifolius*. Suitable environments for *R. longifolius* will decrease if river tributaries and wet lands are claimed by construction works. Therefore, to achieve both conservation and tourism resources, further investigation and careful development plans of the ecosystem are required.

## Materials and Methods

### Study site

This study was done at Kamikochi in Chubu-Sangaku National Park, central Japan (N36°14′57″, E137°38′15″, 1500 m a.s.l., Figure S1). The annual mean precipitation was 2744 mm during 1980–2010. Although temperature was not recorded at Kamikochi, the annual mean temperature was 5.7 °C, estimated from the nearest weather station in Nagawa (1068 m a.s.l.) using the standard lapse rate (−0.6 °C for +100 m altitude). Mean monthly temperatures of the coldest month (January) and the hottest month (August) were −6.2 °C and 17.9 °C, respectively.

Although the vegetation around Kamikochi was natural forest, vehicle roads were along both sides of the Azusa River (Figure S1). The vegetation at 1500 m a.s.l. was the transitional zone between montane deciduous broad-leaved forests and subalpine coniferous forests. Dominant tree species were conifers *Abies homolepis* Siebold et Zucc., *Tsuga diversifolia* (Maxim.) Mast., *Larix kaempferi* (Lamb.) Cariére, and deciduous broad-leaved trees *Ulmus davidiana* Planch. var. *japonica* (Rehder) Nakai, *Betula ermanii* Cham., *Chosenia arbutifolia* (Pall.) A. K. Skvortsov, *Alnus hirsute* Turcz.

### Field methods

Many individuals of *R. obtusifolius*, *R. longifolius* and the hybrids were distributed near the tourist accommodation at Kamikochi Spa and Myojin (Figure S1)^14^. Therefore, this study was done at these two sites. One plot (23 × 13 m) and two plots (22 × 30 m, 8 × 30 m) were established at Kamikochi Spa (Site 1) and Myojin (Site 2), respectively. These plots included various water habitat conditions from dry roadsides to the river tributary.

Individuals of the genus *Rumex* at the three plots were classified into *R. obtusifolius*, *R. longifolius* and interspecific hybrids based on the morphological traits. The most effective identification key for the genus *Rumex* is based on morphological features of completely mature fruits^26^,^36^. A fruit of *R. obtusifolius* has small valves with a tubercle and spines on the margin, while the fruit of *R. longifolius* has large valves with an entire margin and is without a tubercle^22^. Although the fruit size of the hybrids was intermediate between *R. obtusifolius* and *R. longifolius*, the variation was large (our personal observation). The fruit of hybrids has valves with a small tubercle and marginal teeth. Therefore, we identified reproductive individuals of the genus *Rumex* based on the morphological traits of mature fruits.

Leaves and stems of *R. obtusifolius* are reddish and have hairs on the leaf abaxial side, unlike *R. longifolius*^17^,^21^,^22^. Although presence or absence of basal leaves is not a taxonomic key for mature *R. obtusifolius* and *R. longifolius*^22^, basal leaves were present and absent for many reproductive individuals of *R. obtusifolius* and *R. longifolius*, respectively, at our study site. Therefore, we measured qualitative morphological traits presence or absence of reddish color on stem and leaves, basal leaves and hairs on the leaf abaxial side for the three taxa. We also measured stem height and leaf area of the largest leaf of a plant as quantitative morphological traits for all reproductive individuals at the three plots in Sites 1 and 2. The leaf area was estimated by fitting the leaf length and the width to the formula of an ellipse.

Leaves were collected from 40 reproductive individuals of the three taxa at the three plots in Sites 1 and 2 for genetic analyses (described later). In addition to Sites 1 and 2, leaves were sampled from 32 individuals of the three taxa at Myojin (Site 3), Taisho Pond (Site 4) and Tokusawa (Site 5) for genetic analyses (Figure S1). Species were identified mainly by the valves of mature fruits at Sites 1 and 2. However, most individuals were non-reproductive individuals at Sites 3–5, except for *R. obtusifolius* at Site 4. Therefore, we identified species, based on the other morphological traits (presence or absence of reddish color on leaves and stems and hairs on the abaxial side of leaves), for non-reproductive individuals. Collected leaves were dried and were stored in silica gel until DNA extraction.

Water habitat conditions of all individuals at the three plots in Sites 1 and 2 were classified into river tributary, wet land, meadow, roadside. The difference between the river tributary and wet land was whether the base of a plant stem was under water (river tributary) or not (wet land). Wet lands were located at the periphery of river tributaries. Soils of the meadow were wetter than the bare ground of the roadside.

*R. obtusifolius*, *R. longifolius* and the hybrid individuals were compared by using the χ^2^-test for habitat conditions and the three qualitative morphological traits, and by using the non-parametric Kruskal-Wallis test for the two quantitative morphological traits.

### Germination tests of pollen and seeds

Ten individual plants were used to test germination rates of pollen for each of *R. obtusifolius*, *R. longifolius* and the interspecific hybrids. The germination rate of pollen was estimated by *in vitro* pollen germination analysis. Germinated pollen was assessed after dusting fresh pollen onto a germination medium composed of agar (1%) and sucrose (10%) in petri dishes. The petri dishes were kept at 20 °C in an incubator before the assessment of pollen germination. Total numbers of pollen grains examined were 1428, 795 and 756 for *R. obtusifolius*, *R. longifolius* and the interspecific hybrids, respectively. A significant difference in pollen germination rate (%) among the three taxa was tested by the 95% confidence intervals generated using a 1000 iteration bootstrap technique^37^.

Seed germination tests were done by placing 100 seeds of each species on two filter papers in a petri dish. To examine the relationship between seed germination and water conditions for each species, we used water conditions of complete flooding (total immersion in water), light flooding (half immersed in water) and no flooding. Flood conditions were simulated by shallow flooding with deionized water in the petri dish. Water levels in the petri dishes were kept at 1 cm and 0.5 cm for the complete and light flooding conditions, respectively. A nylon-meshed cloth above seeds prevented seeds from floating in the complete flooding condition. Two filter papers in the petri dish were kept in a wet condition in the no flooding condition. Radicle emergence was taken as proof of germination. The temperature was set at 25 °C during the 12 hours day period and 10 °C night temperature in an incubator. The experiments lasted 28 days. The number of replicates of the germination tests was two for each water condition for each of the three taxa. Significant differences in seed germination rate (%) among the three water conditions and among the three taxa were tested by the 95% confidence intervals generated using a 1000 iteration bootstrap technique^37^.

### Amplified fragment length polymorphism

Approximately 200 μl DNA were obtained from a leaf sample of 5 mm square by using the DNeasy Plant mini Kit (QIAGEN Inc., Venlo, Netherlands) for AFLP analysis^38^. Ten μl of DNA were digested for 1.5 h at 37 °C with 0.25 μl *Eco*RI (100 U/μl) and 0.1 μl *Mse*I (50 U/μl) in a 25 μl reaction volume that included 2.5 μl of 10 × NE Buffer and 0.25 μl of 100 × BSA (10 mg/ml). Digested DNA was ligated with double-stranded adaptors at 20 °C overnight. After 2 min at 72 °C, pre-selective polymerase chain reaction (PCR) amplification was done for 20 cycles of denaturation (20 s at 94 °C), annealing (30 s at 56 °C) and extension (2 min at 72 °C) using a primer pair with one additional nucleotide on each restriction enzyme (*Mse*I-C/*Eco*RI-A). The pre-amplification product was diluted 1:20 with TE 0.1 buffer and was used for selective amplification. Selective amplifications were then done with fluorescence-labeled primer combinations *Eco*RI-ACC (NED)/*Mse*I-CTA and *Eco*RI (FAM)-ACT/*Mse*I-CAT. After 2 min at 94 °C, 30 cycles of amplification were done under the following conditions: denaturation for 20 s at 94 °C; annealing for 30 s; extension for 2 min at 72 °C. The annealing temperature of the first cycle was 66 °C, followed by a decrease in temperature (1 °C in each cycle) in the following 10 cycles, and finally it was maintained at 56 °C for the remaining 19 cycles. A LifeECO Thermal Cycler (Bioer Technology Co., Ltd., Binjiang, China) was used with the AFLP Amplification Core Mix (Applied Biosystems, Foster City, CA, USA) for both pre-selective and selective amplifications. The AFLP fragments were detected by using an ABI Prism 3130 automated sequencer (Applied Biosystems) and Gene Mapper software version 4.0 (Applied Biosystems). We used the loci that minimum peak height was greater than 50, and the resulting data set was transformed into a binary matrix for further statistical analysis.

### Data analysis

The population genetic structure was inferred using the Markov chain Monte Carlo (MCMC) and the Bayesian clustering algorithms implemented by the Structure software (version 2.3.4)^39^. Structure analysis was done with a burn-in period of 50,000 and 50,000 subsequent MCMC steps. We tested for the number of clusters (*K*) from 1 to 10 with 10 replicates for each *K*. The ad-hoc statistic ∆*K* was used to detect the most likely number of populations^40^. The *∆K* statistic was based on the second order rate of change of the posterior probability of the data between successive *K* values over 10 replicates. The highest *∆K* was the best *K* value^40^. In addition to the Structure analysis, we used PCoA with binary data obtained from the GeneMapper for the clustering of individuals. Cluster analysis was also performed, using the complete linkage method, to identify clusters in PCoA.

We used the Bayesian statistical approach, NewHybrids software (version 1.1)^41^, to distinguish hybrids from purebred individuals. NewHybrids software uses an MCMC algorithm. We did the NewHybrids analysis with a burn-in period of 5,000 and 100,000 subsequent MCMC steps, and then the posterior probability was computed for each of the six classes: the two parental species, F1 and F2 generations, and backcrosses to each parental class.

## Additional Information

**How to cite this article**: Takahashi, K. and Hanyu, M. Hybridization between alien species *Rumex obtusifolius* and closely related native vulnerable species *R. longifolius* in a mountain tourist destination. *Sci. Rep.* **5**, 13898; doi: 10.1038/srep13898 (2015).

## Supplementary Material

Supplementary Information

## Figures and Tables

**Table 1:** Comparison of qualitative morphological traits of presence or absence of hairs on the leaf abaxial side, basal leaves and reddish leaf and stem of reproductive individuals among *Rumex obtusifolius*, *R. longifolius* and their hybrids.

Species	Hairs on leaf abaxial side		Reddish leaf and stem		Basal leaves	
	Absence	Presence	Absence	Presence	Absence	Presence
*R. longifolius*	56	0	41	15	9	47
*R. obtusifolius*	1	108	2	107	109	0
Hybrid	0	28	0	28	24	4
χ^2^	188.2^***^		118.2^***^		136.5^***^	

Species was identified, based on morphological traits of mature fruits. The values are the numbers of individuals of presence and absence of the three characteristics and their χ^2^ values (d.f. = 2).

***P < 0.001.
