# Supplementary material for: Hybridization between alien species Rumex obtusifolius and closely related native vulnerable species R. longifolius in a mountain tourist destination
Source: Sci Rep. 2015 Sep 10;5:13898. doi: 10.1038/srep13898 (PMC4564823; doi:10.1038/srep13898)
Supplement: Supplementary Information [file srep13898-s1.pdf]

Supplementary Information:

Hybridization between alien species *Rumex obtusifolius* and closely related native vulnerable species *R. longifolius* in a mountain tourist destination

Koichi Takahashi & Masaaki Hanyu

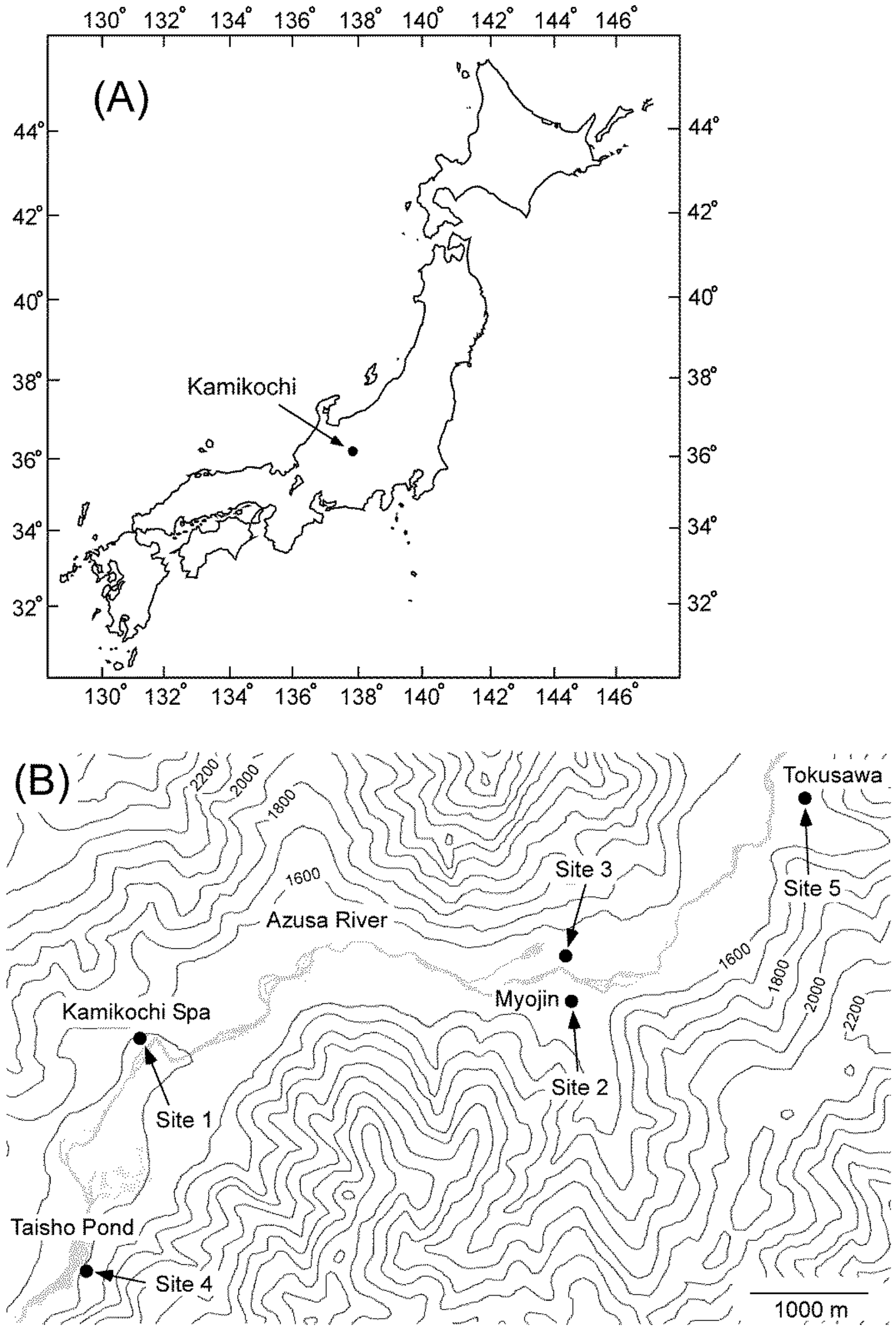

Figure S1: (A) Location of Kamikochi in central Japan. (B) Sampling was done at five sites in Kamikochi. Contour lines are at 100 m intervals. The maps were created by processing the map prepared by Geospatial Information Authority of Japan, using Canvas 7.

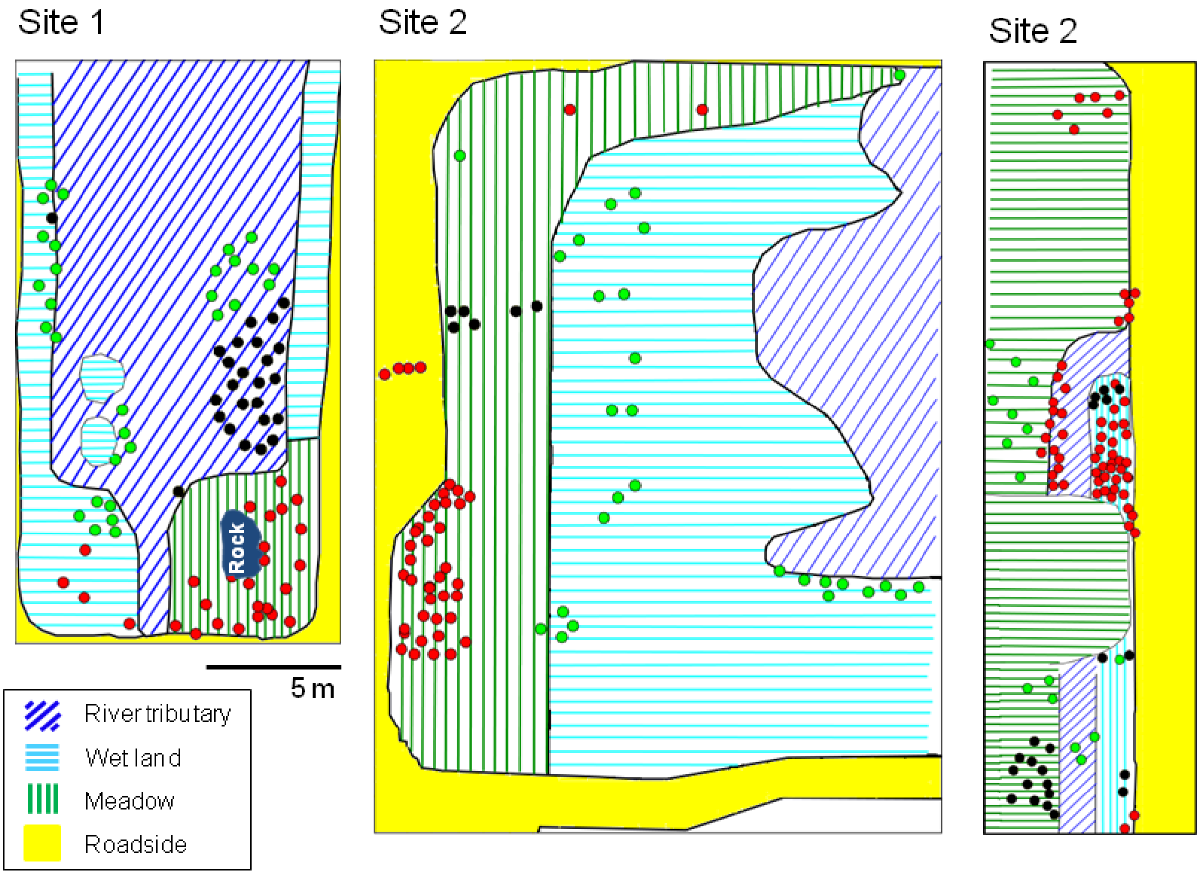

Figure S2: Spatial distribution patterns of *Rumex obtusifolius* (●), *R. longifolius* (●) and the hybrid (●) at one plot in Site 1 and two plots in Site 2.

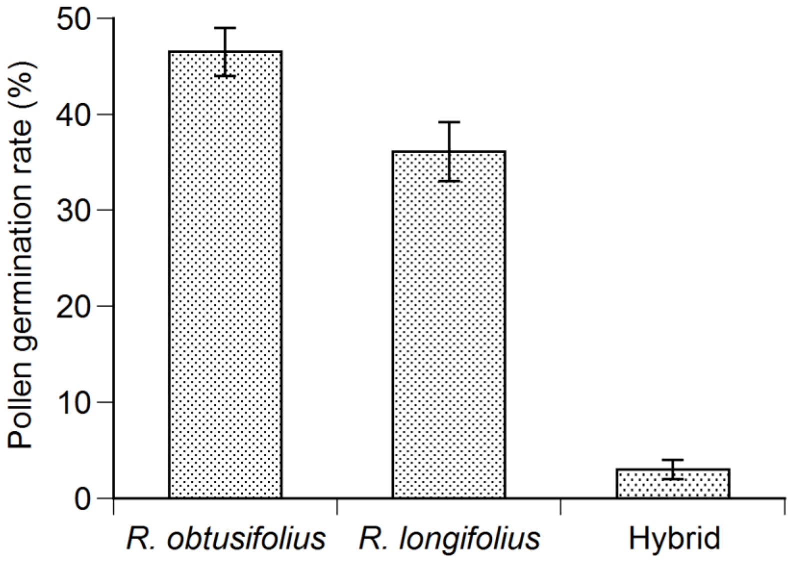

Figure S3: Pollen germination rates (%) of *Rumex obtusifolius*, *R. longifolius* and the hybrid at 95% confidence intervals.

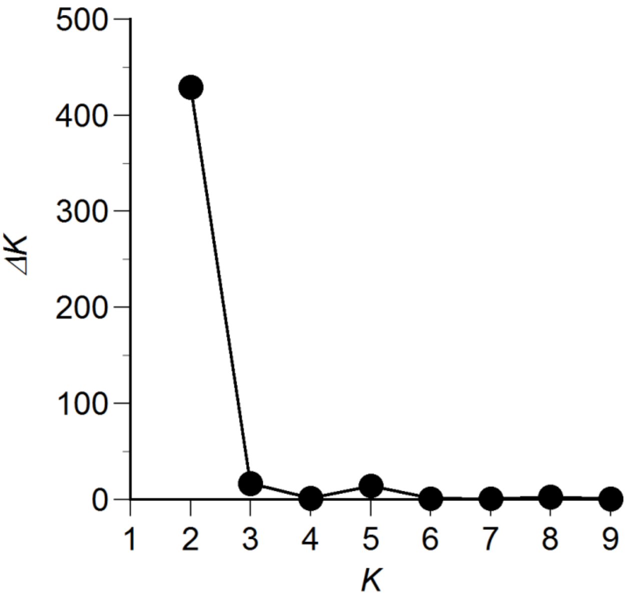

Figure S4: Plot of Δ*K* for each *K* value was calculated, where *K* is the number of clusters. The mean of ten independent runs for each value of *K* ranged from 2 to 9.
